# Clinical presentation, diagnostic findings and outcome of dogs undergoing surgical resection for intracranial meningioma: 101 dogs

**DOI:** 10.1186/s12917-022-03182-y

**Published:** 2022-03-07

**Authors:** Alexander K. Forward, Holger Andreas Volk, Giunio Bruto Cherubini, Tom Harcourt-Brown, Ioannis N. Plessas, Laurent Garosi, Steven De Decker

**Affiliations:** 1 Davies Veterinary Specialists, Higham Gobion, Hitchin, SG5 3HR UK; 2 Department of Small Animal Medicine and Surgery, University of Veterinary Medicine Hannover, 30559 Hannover, Germany; 3 Dick White Referrals, Station Farm, London Road, Six Mile Bottom, Cambridgeshire, CB8 0UH UK; 4 Langford Small Animal Referral Hospital, Langford House, Langford, Bristol, BS40 5DU UK; 5 Vet Oracle Teleradiology, Bedford, UK; 6 Department of Clinical Science and Services, Royal Veterinary College, University of London, North Mymms, AL9 7TA UK

**Keywords:** Meningioma, Craniotomy, Hydroxyurea, Ultrasonic aspirator, Intracranial, Survival, Transfrontal, Rostrotentorial

## Abstract

**Background:**

Meningioma is the most common primary brain neoplasm in dogs. Further information is required regarding the expected long-term prognosis of dogs following the surgical resection of an intracranial meningioma together with the influence of adjunctive therapies. Whilst there have been several studies reporting the long-term outcome of intracranial meningioma resection following surgery alone, surgery with the use of an ultrasonic aspirator, surgery combined with radiotherapy and surgery combined with the addition of hydroxyurea, it is currently unclear which type of adjunctive therapy is associated with the most favourable outcomes. The objective of this study is to describe the presentation and outcome of dogs undergoing surgery for the resection of an intracranial meningioma and the effect of clinical factors, adjunctive therapies and meningioma histopathological subtype on the long-term outcome.

**Results:**

A hundred and one dogs that had intracranial surgery for meningioma resection were investigated from four referral centres. 94% of dogs survived to hospital discharge with a median survival time of 386 days. Approximately 50% of dogs survived for less than a year, 25% survived between 1 and 2 years, 15% survived between 2 and 3 years and 10% survived for greater than 3 years following discharge from hospital. One or more adjunctive therapies were used in 75 dogs and the analysis of the data did not reveal a clear benefit of a specific type of adjunctive therapy. Those dogs that had a transfrontal approach had a significantly reduced survival time (MST 184 days) compared to those dogs that had a rostrotentorial approach (MST 646 days; *p* < 0.05). There was no association between meningioma subtype and survival time.

**Conclusions:**

This study did not identify a clear benefit of a specific type of adjunctive therapy on the survival time. Dogs that had a transfrontal approach had a significantly reduced survival time. Intracranial surgery for meningioma resection offers an excellent prognosis for survival to discharge from hospital with a median long term survival time of 386 days.

## Background

Meningioma is the most common primary brain neoplasm in dogs [1]. The decision for both the owner and clinician to perform intracranial surgery for the resection of a meningioma is multifactorial and can be difficult. Whilst previous literature has investigated the survival times for the treatment of such cases [2–4], further information is required regarding the expected long-term prognosis for these dogs to allow informed decisions regarding their treatment.

There have been several studies reporting the long-term outcome of intracranial meningioma resection following surgery alone, surgery with the use of an ultrasonic aspirator, surgery combined with radiotherapy and surgery combined with the addition of hydroxyurea [2–7]. Although there are indications that surgery combined with adjunctive therapy is associated with a prolonged survival time [2, 4], it is currently unclear which type of adjunctive therapy is associated with the most favourable outcomes.

The primary aim of this study was to evaluate the presentation, treatment and long-term outcome of dogs undergoing surgical resection of intracranial meningiomas and the influence of meningioma location and surgical approach, commonly used adjunctive therapies and histopathological subtype on their long-term outcome. We hypothesized that meningiomas that are surgically resected with the use of an ultrasonic aspirator followed by radiotherapy offer the longest survival times.

## Results

A hundred and one dogs with a median age of 118 months (IQR: 96–132) were included in this study. There were 58 males (39 neutered) and 43 females (41 neutered). Breeds included German Shepherd (*n* = 15), Labrador Retriever (*n* = 15), Cross breed (*n* = 12), Boxer (*n* = 13), West Highland White Terrier (*n* = 4), Springer Spaniel (*n* = 4), Golden Retriever (*n* = 5), English Cocker Spaniel (*n* = 3), Jack Russell Terrier (*n* = 3), Staffordshire Bull Terrier (*n* = 4), Border Collie (*n* = 4), Weimaraner (*n* = 2), Miniature Poodle (*n* = 2), Rhodesian Ridgeback (*n* = 2), Airedale terrier (*n* = 1), Cavalier King Charles Spaniel (*n* = 1), Pointer (*n* = 1), Alaskan Malamute (*n* = 1), Dalmatian (*n* = 1), Stabyhoun (*n* = 1), Greyhound (*n* = 1) Newfoundland (*n* = 1), Cairn Terrier (*n* = 1), Irish Setter (*n* = 1), Sheltie (*n* = 1), Sharpei (*n* = 1) and a French Bulldog (*n* = 1).

Ninety-one percent (92/101) of dogs had a history of seizure activity and 49 % (49/101) of dogs had neurological abnormalities detected on examination at the time of presentation. Reported neurological abnormalities included menace response deficits (*n* = 17), other cranial nerve deficits (*n* = 12), ataxia (*n* = 26), an abnormal mentation (*n* = 26), reduced postural reactions (*n* = 23), a head tilt (*n* = 7), head pressing (*n* = 2), circling (*n* = 2) and a low head carriage/cervical pain (*n* = 2). Two dogs were neurologically normal at the time of examination with no history of seizure activity as the meningiomas were incidentally identified during advanced imaging of the bullae.

All dogs underwent magnetic resonance imaging (MRI) of the brain with the primary location of the meningiomas as follows: olfactory lobe (33/101), frontal lobe (36/101), parietal lobe (10/101), temporal lobe (9/101), occipital lobe (6/101) and the cerebellum (7/101). Forty-nine percent (50/101) of dogs had no further imaging performed for staging, 42% (42/101) had both thoracic and abdominal imaging and 9% (9/101) had one of either thoracic or abdominal imaging performed. In 12% (6/51) of dogs that had additional imaging, one or more abnormalities were detected. Two dogs had enlarged adrenal glands identified, two had enlarged retropharyngeal lymph nodes, one had a colonic lesion, one had pulmonic nodules and one had a testicular mass identified. No further investigation was performed for any of these lesions apart from the testicular mass which was diagnosed as an interstitial cell tumour.

All dogs subsequently had surgery with 57% (58/101) having a transfrontal approach, 36% (36/101) having a rostrotentorial approach and 7% (7/101) having a suboccipital approach. Eighty one percent (82/101) of dogs received pre-operative glucocorticoids and 87% (88/101) received post-operative glucocorticoids. Ninety one percent (92/101) of dogs were administered anti-epileptic medication with 71% (72/101) receiving phenobarbital, 5% (5/101) receiving levetiracetam, 1% (1/101) receiving potassium bromide and 2% (2/101) receiving imepitoin as sole therapies. Twelve percent (12/101) received a combination of two of the aforementioned medications.

Twenty-six percent of dogs (26/101) had surgery without the utilisation of an adjunctive therapy with 74% (75/101) of dogs having at least one form of adjunctive therapy used. Thirty two percent of dogs (32/101) had a single adjunctive therapy added, 31% (31/101) had 2 adjunctive therapies, 11% (11/101) had 3 adjunctive therapies and one dog (1/101) had all four adjunctive therapies utilised. The most commonly used adjunctive therapy was the utilisation of an ultrasonic aspirator in 56% (57/101) of dogs, followed by the administration of post-operative hydroxyurea in 41% (41/101) of dogs, intraoperative topical chemotherapy in 22% (22/101) of dogs and post-operative radiotherapy in 11% (11/101) of dogs. For those dogs receiving hydroxyurea, the duration of treatment varied with the shortest being 2 weeks due to the development of methemoglobinaemia and the longest treatment course being 844 days which was up to the point of death. The only potential side effect of hydroxyurea identified was the aforementioned methemoglobinaemia in one dog. Eleven dogs (11/101) were sent for post-operative radiotherapy with only nine dogs completing their courses. Two dogs died in the middle of their treatment course with one developing sepsis secondary to an intravenous infection and in the other the cause of death was unknown.

All of the resected meningiomas were sent for histopathology with the different meningioma subtypes listed in Table 1. The meningotheliomatous (28/101) and transitional (23/101) histopathological subtypes were the most common to be identified, accounting for more than 50% of the total number of meningiomas when combined. The median survival time (MST) for the dogs that survived to discharge were calculated for the different histopathological subtypes and are presented in Table 1 with no significant association between meningioma subtype and survival time.

**Table 1:** Meningioma histological classification of all 101 dogs together with the median survival times

Meningioma subtype *(total number of cases: 101)*	Median survival time (days) following hospital discharge *(number of dogs)*	95% Confidence Interval	
		*Lower Bound*	*Upper Bound*
Papillary (9) *	1079 (7)	134.3	2023.7
*Rhabdoid (1)*	572 (1)	.	.
Angiomatous (angioblastic) (5) *	454 (5)	95.4	812.6
Fibrous (fibroblastic) (4) *	417 (3)	.000	925.906
Meningotheliomatous (28) *	386 (25)	184.5	587.5
*Vacuolar (1)*	385 (1)	.	.
Psammomatous (12) *	363 (10)	118.2	607.8
Transitional (mixed) (23) *	307 (20)	0.0	850.5
*Unknown (10)*	252 (9)	82.5	421.5
Anaplastic (malignant) (2) *	123 (2)	.	.
*Cystic (4)*	99 (4)	.000	443.9
*Choroid (1)*	–	–	–
*Atypical (1)*	–	–	–

*Legend*: The median survival time of those dogs that survived to discharge is detailed depending on the histopathological subtype. Those with a * are those listed in the histological classification of tumours of the nervous system of domestic animals (WHO): Tumours of the meningothelial cells [8]. The others are subtypes that were later identified independently in dogs following the WHO classification system

Sixteen percent (16/101) of dogs experienced a postoperative complication whilst hospitalised. The most common complication was a post-operative aspiration pneumonia which occurred in 6% (6/101) of dogs following surgery. Other non-fatal complications reported were a non-haemorrhagic nasal discharge (*n* = 2), a subcutaneous emphysema (*n* = 1) and a surgical site seroma (*n* = 1). Six percent (6/101) of dogs did not survive to the point of hospital discharge due to complications including a worsening neurological deterioration resulting in euthanasia (*n* = 2), post-operative cardiac arrest (*n* = 2), suspected malignant hyperthermia syndrome (*n* = 1) and respiratory arrest (*n* = 1).

**Fig. 1:**
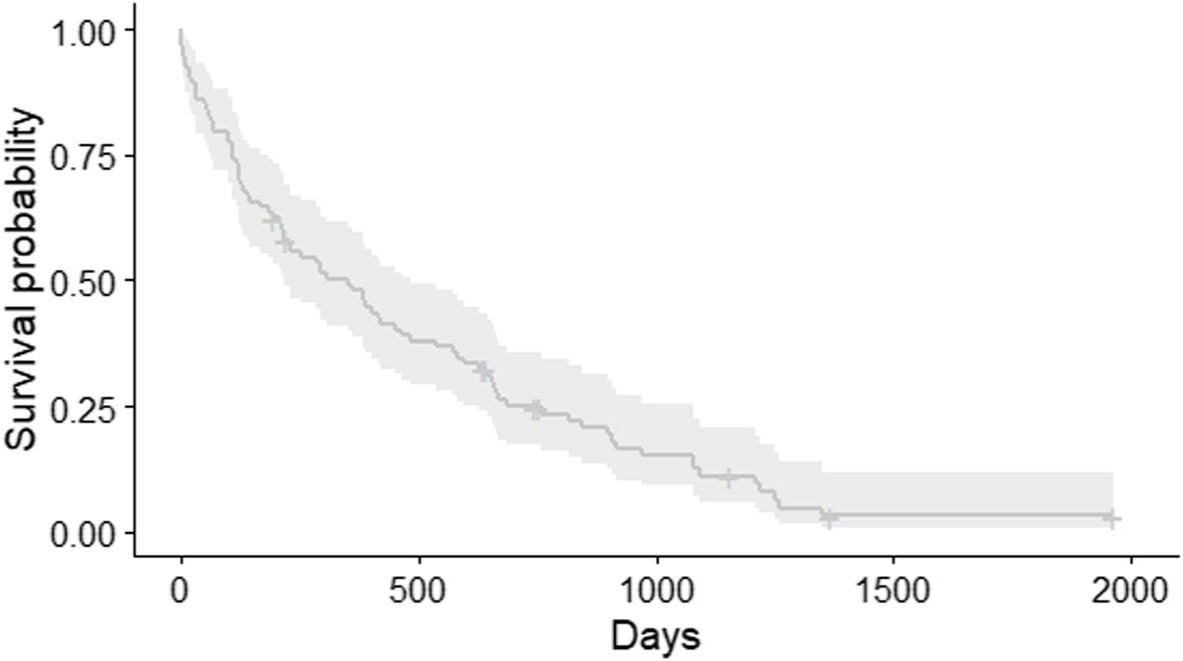
Kaplan–Meier survival function for death due to all causes in 101 dogs that underwent surgical resection of an intracranial meningioma. Legend: Kaplan–Meier survival function with 95% confidence band. The plus symbols represent censored observations

The median survival time using all-cause mortality for all dogs that underwent intracranial surgery for the resection of a meningioma was 353 days ((95% CI 218–485) Fig. 1) with survival proportions at 6 months of 0.64 (0.55–0.75), at 1 year of 0.48 (0.39–0.59), and at 3 years of 0.11 (0.06–0.21).

Ninety-four percent (95/101) of dogs survived to hospital discharge. Eight dogs were lost-to-follow-up following discharge and were not analysed leaving 87 dogs with long term follow up. Ten of these dogs are still alive at the time of writing. Of the 77 dogs that did not survive, 66% (51/77) died due to neurological deterioration (worsening seizure activity or progressive neurological signs), 17% (13/77) had a reported non-neurological cause of death and for 18% (13/77), the cause of death was not known. Where the cause of death was known, 80% (51/64) died due to a neurological deterioration with the remainder dying from non-neurological causes. Two dogs had repeat surgeries for meningioma regrowth following a neurological deterioration at 664 days and 1703 days respectively following the original surgery, with the latter still alive at the time of writing. Three dogs developed and were treated for a post-operative sinusitis two of which had a positive culture for aspergillosis. For the latter, one developed aspergillosis 371 days following the original surgery and the other developed it 250 days following a repeat surgery following meningioma regrowth.

Fifty-one percent (44/87) of dogs did not survive greater than a year following discharge, 26% (23/87) of dogs died between 1 and 2 years following discharge, 14% (12/87) of dogs died between 2 and 3 years and 9% of dogs lived for greater than 3 years following discharge from hospital. Ten percent (9/87) of dogs survived less than 2 months following hospital discharge due to worsening seizures and/or a neurological deterioration (*n* = 5), development of tension pneumocephalus (*n* = 2) and the remaining two had an unknown cause of death (*n* = 2).

The median survival time using all-cause mortality for dogs that were discharged from hospital was 386 days (95% CI 271.4–500.6) with survival proportions at 6 months of 0.70 (0.60–0.81), at 1 year of 0.55 (0.45–0.68), and at 3 years of 0.24 (0.14–0.39). The median survival time using cause specific mortality for dogs that were discharged from hospital that died due to a neurological deterioration was 421 days (95% CI 287–687) with survival proportions at 6 months of 0.69 (0.60–0.79), at 1 year of 0.51 (0.42–0.63), and at 3 years of 0.12 (0.06–0.22).

There was no significant difference in the survival time between the different treatment groups listed in Table 2.

**Table 2:** The median survival time of dogs following discharge from hospital depending on the adjunctive therapy utilised and the surgical approach

Adjunctive therapy				*Number of dogs*	*Median survival time (days)*	95% Confidence Interval	
*Ultrasonic aspirator*	*Hydroxyurea*	*Radiotherapy*	*Topical Chemotherapy*			*Lower Bound*	*Upper bound*
*	*	*	*	1	1079	–	–
*		*		2	918	–	–
	*			6	626	121.9	1130.1
*				18	421	155.9	686.1
	*	*		2	417	–	–
				21	353	246.8	459.2
*	*			17	293	0.0	801.9
		*		4	232	87.0	377.0
*	*		*	10	166	0.0	558.0
	*		*	3	144	84.8	203.2
*			*	3	99	0	206.2
**Surgical approach**							
*Sub-occipital*				5	898	336.5	1459.5
*Rostrotentorial*				33	646	327.4	964.6
*Transfrontal*				49	184	90.2	277.8
**Total number of dogs**				87	386	271.4	500.6

*Legend*: * indicates the utilisation of that particular adjunctive therapy

The median survival time of those dogs that had surgery alone without the addition of any adjunctive therapy was 353 days (95% CI 246.8–459.2) compared to dogs that had one or more of any form of adjunctive therapy utilised having a median survival time of 403 days (95% CI 220–586.0), with no significant difference in survival between these two groups. The median survival time of all dogs that received topical chemotherapy (in combination with other adjunctive therapies) was 166 days (95% CI 55.7–276.4) whilst the median survival time of dogs receiving any of the other adjunctive therapies (apart from topical chemotherapy) was 454 days (95% CI 258.7–649.3), which was significantly different (*p* < 0.05). When this latter group was compared to those dogs that had surgery without any adjunctive therapy, there was no significant difference in survival.

**Fig. 2:**
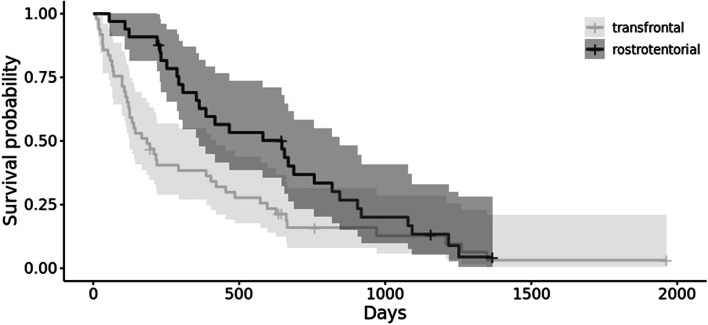
Kaplan–Meier survival functions comparing transfrontal and rostrotentorial surgical approaches for meningioma resection. Legend: Kaplan–Meier survival functions with 95% confidence bands for death due to all causes in dogs that survived to hospital discharge following the surgical resection of an intracranial meningioma comparing transfrontal and rostrotentorial surgical approaches. The plus symbols represent censored observations

Of the 87 dogs with long term follow up, 49 had a transfrontal approach with a median survival time of 184 days (95% CI 90.2–277.8), 32 had a rostro-tentorial approach with a median survival time of 646 days (95% CI 317.2–974.8), 5 dogs had a suboccipital approach with a median survival time of 898 days (95% CI 336.5–1459.5) and 1 dog had a combined transfrontal and rostrotentorial approach with a survival time of 29 days. When these groups were compared, those dogs having a transfrontal approach had a significantly reduced survival time compared to those that had a rostrotentorial surgical approach ((*p* < 0.05) Fig. 2). There was no difference between those dogs that had a suboccipital approach when compared to either of the other surgical approaches.

## Discussion

This study provides additional data on the signalment, neuroanatomical location, histopathological subtype and long-term outcome following the surgical resection of intracranial meningiomas. Although this data is available for cats following the recent publication of a large multicentre study [9], there remains limited information available for dogs. The results of this study illustrate the variability and differing combinations of adjunctive treatment protocols currently utilised in dogs following the surgical resection of an intracranial meningioma. Whilst there was no significant difference in survival time with the addition of any specific adjunctive therapy, it highlights that surgery for meningioma resection offers an excellent prognosis for hospital discharge and a median long-term survival of 386 days.

In human medicine, intracranial meningiomas are most commonly treated via their primary excision, which is also true of cats in veterinary medicine [10]. In cats, we often think of the surgical excision of an intracranial meningioma as curative, with a reported median survival time of 37 months and the majority of cats eventually dying of causes unrelated to the meningioma [9]. Unlike dogs, meningiomas in cats are often better defined and less invasive [8, 9, 11], and hence further adjunctive therapy following their excision is not typically necessary. However dogs have a larger percentage of higher grade meningiomas (atypical grade II) when compared to cats and humans [11]. This higher incidence of atypical (grade II) meningiomas in dogs and their more invasive nature might account for their poorer therapeutic response when compared to humans and cats [11]. Because of this, additional adjunctive therapies are often used following the surgical excision of meningiomas in dogs in order to try and augment their survival time.

The MST for dogs that had standard cytoreductive surgery alone without the addition of any of the adjunctive therapies was 353 days compared to dogs that had one or more of any form of adjunctive therapy utilised having a median survival time of 403 days, with no significant difference in survival between these two groups. Whilst hugely variable, the average MST for dogs that had standard cytoreductive surgery in previous studies was approximately 10 months (range 7 months − 16.5 months) [1, 4, 12].

An ultrasonic aspirator was the most common adjunctive therapy utilised with more than 50% of all dogs in this study having it used. For those dogs where an ultrasonic aspirator was used during surgery without the addition of any other adjunctive treatments (*n* = 18), the MST was 421 days and not statistically different to those that had surgery alone. Ultrasonic aspirators are widely used in human neurosurgery for the resection of meningiomas and allow preferential ablation of tissue with a higher water content such as neoplastic tissue [2, 13]. Previous studies examining the use of aspirators for meningioma resection in dogs reported a MST of 1274 days [2, 14, 15]. However, this MST was based on the analysis of approximately three surviving dogs only [2, 16]. Another study used an aspirator in 23 dogs for the excision of intracranial meningiomas with 16 of these dogs surviving for more than 2 years following surgery [17]. However, an intraoperative MRI was also used in this study which might also have contributed to the extended survival times reported [17].

Hydroxyurea is an antimetabolite that specifically affects the S stage of the cell cycle and has been suggested as a chemotherapeutic agent for the management of meningiomas [3]. The administration of hydroxyurea was the second most commonly used adjunctive therapy with 41% (41/101) of dogs being administered it. For those dogs that had hydroxyurea administered without the addition of any other adjunctive treatments (*n* = 6), the MST was 626 days and not statistically different to those that had surgery alone. To date, there have been a few reports of the use of hydroxyurea in dogs following meningioma resection and whilst many dogs in this study received hydroxyurea, very few had it as the sole adjunctive therapy making any meaningful comparisons difficult [5–7]. It is worth noting that the dose, frequency of administration and treatment length of hydroxyurea varied widely between dogs making it difficult to allow accurate comparisons. In humans, hydroxyurea has been shown to increase progression-free survival following incomplete resection of meningiomas and hence this treatment is potentially worth investigating further in the future [18, 19].

Eleven dogs were administered post-operative radiotherapy with only 4 dogs receiving it as the sole adjunctive therapy. Two of these are still alive at the time of writing and two didn’t complete the full radiotherapy course as they were euthanised before completion. It is interesting that this was the least utilised of the four adjunctive therapies particularly given it is the one with the strongest evidence base compared to other types of adjunctive therapy [4, 20, 21]. Reasons for this might include the cost, perceived invasiveness of multiple general anaesthetics, prolonged periods of time away from home and the fact that it was not available at any of the treatment centres that performed the surgery. The MST in this study is far below that which has been previously reported in the literature which ranges from 16 to 30 months for dogs having had surgical resection and post-operative radiotherapy [4, 20, 21]. Given the small number of dogs in this group, the variation in radiotherapy protocols received and the fact that two didn’t complete their course and two are still alive, the MST reported in this study is likely not reflective of the true benefit of post-operative radiotherapy. In humans, radiotherapy as a sole treatment or following incomplete surgical resection of meningiomas has been shown to be beneficial with 5-year progression-free survival rates of 80–100% for both benign and atypical tumours [22, 23].

One centre used intraoperative topical chemotherapy as a clinical trial up until 2015 in the form of methotrexate and cytosine arabinoside following the removal of the meningioma. It was never used as the sole adjunctive therapy and was always in combination with one of those previously discussed. The MST of all dogs that received topical chemotherapy (in combination with other adjunctive therapies) was 166 days whilst the median survival time of dogs receiving any of the other adjunctive therapies (apart from topical chemotherapy) was 454 days (95% CI 258.7–649.3), which was significantly different (*p* < 0.05). There is a report of the use of this combination of topical chemotherapy in the veterinary literature with one previous paper reporting its use following the excision of an intracranial inflammatory fibrosarcoma [24]. The reduced survival time for those dogs that received this topical chemotherapy in this study could be due to the direct action of these agents themselves or potentially for different reasons but based on these results, it cannot be recommended as an adjunctive treatment following the surgical excision of an intracranial meningioma.

The most common breeds in this study included Labradors, German Shepherds and Boxers with no sex predilection identified, which is consistent with previous studies [2, 4, 11, 25]. The most common presenting clinical complaint was seizure activity with over 90% of dogs having had documented seizures at the time of presentation. This is different to cats where seizures are less common as a presenting sign seen only in approximately 14–25% of cats [26–28]. Over 92% of the meningiomas resected were in the forebrain with almost 70% of the meningiomas being associated with either the olfactory lobe or frontal lobe, consistent with previous reports [11, 29]. This might be reflective of our inclusion criteria as it is possible our study selected for cases that had meningiomas in locations thought to be more surgically accessible such as those in the frontal and olfactory lobes. Fifty percent of dogs in this study had extracranial imaging performed as part of staging and none had evidence of extra-cranial meningioma metastases, possibly suggesting that extracranial imaging might not be worthwhile in intracranial meningioma cases. However this also might be reflective of our inclusion criteria as it is possible our study selected for cases that had no evidence of extra-cranial metastases before intracranial surgery was performed. Hence, thoracic and abdominal imaging could still be considered in such cases before performing often expensive and invasive intracranial surgery, since 27% of meningioma patients were found to have an unrelated but potentially clinically relevant form of neoplasia in a post-mortem examination study [29]. Of those in our study that had extracranial imaging performed, 12% had clinically unrelated abnormalities identified with only one confirmed to be neoplastic in nature.

Meningiomas are particularly interesting tumours due to the number of histological subtypes that exist. The meningotheliomatous (28/101) and transitional (23/101) histological subtypes were the most commonly identified in this study, together accounting for greater than 50% of the total number of meningiomas. This is similar to other studies that have also shown these two subtypes to be the most commonly identified in dogs [11, 30, 31]. In cats, the most frequent subtypes identified tend to be the psammomatous and transitional meningiomas with anaplastic meningiomas being very rare [9, 32, 33]. In humans, the anaplastic variant of meningioma is also rare accounting for 1–3% of all meningiomas in people with the most common morphological subtypes in humans being meningothelial, fibroblastic, transitional and psammomatous [34–36]. We found no significant association between long-term survival and histopathological tumour type using the domestic animal WHO tumour classification system [8]. However, a future study using the WHO international histological classification of human meningiomas into grades I, II or III or the molecular subtyping of meningiomas could be interesting in order to ascertain if there is an association between tumour grade or the molecular characterisation and long-term prognosis [37, 38].

Whilst the removal of a brain tumour can be a daunting prospect for both the clinician and owner, this study shows that the survival to hospital discharge following such a procedure is relatively high at almost 95%. Sixteen percent of dogs developed a post-operative complication whilst hospitalised, with the most common being aspiration pneumonia seen in 6% of dogs, which is consistent with previous studies examining intracranial surgery in dogs [39, 40].

Interestingly, those dogs that had a transfrontal surgical approach in this study had a significantly reduced survival time compared to those that had a rostrotentorial approach. Given the many differences already identified in how these patients are treated and hence the possibility of many confounding factors within these two groups, it could be a type 1 error and hence we are cautious as to how much significance to place on this finding. However potential reasons for this difference might include that the rostrotentorial approach affords better access to allow a more complete surgical excision or that the transfrontal approach results in more frequent long term post-operative complications that are life limiting or a possible increased risk of post-operative seizures. Further work is needed to confirm this difference and to ascertain the potential underlying reasons for it.

The limitations of this study are reflective of its retrospective nature with a very heterogenous sample, neurosurgeons of differing experience and a nearly universal lack of quantifiable assessment of the extent of surgical resection (be it surgeon or imaging based). Long term follow up of such cases can be challenging for many reasons and the cause of death with these dogs should be treated with caution as to differentiate the cause of death as neurological due to regrowth or non-neurological in nature is difficult particularly in a mainly geriatric population.

The aim of this study was to detail what is currently being done in the surgical removal of meningiomas in dogs. It encompasses four large referral centres in the UK and due to its sample size, gives a fairly accurate estimation of the long-term survival of dogs following intracranial meningioma resection. This study suggests that whilst adjunctive therapy for such cases is often utilised, there is no clear consensus on which of those described is the most appropriate and reflects the current lack of evidence-based treatment. The results of this study fail to identify the superiority of one type of adjunctive therapy but further work is needed to allow for more meaningful comparisons in the form of prospective studies, which will hopefully allow for superior treatment of this condition in the future.

## Conclusion

The results of this study illustrate the variability and differing combinations of adjunctive treatment protocols currently utilised in dogs following the surgical resection of an intracranial meningioma. Whilst there was no significant difference in survival time with the addition of any specific adjunctive therapy, it highlights that surgery for meningioma resection offers an excellent prognosis for hospital discharge and a median long-term survival of 386 days. Those dogs that had a transfrontal approach had a significantly reduced survival time (MST 184 days) compared to those dogs that had a rostrotentorial approach (MST 646 days; *p* < 0.05) and there was no association between meningioma subtype and survival time.

## Materials and methods

Medical records from four referral centres (2 university teaching hospitals and 2 private referral centres) were searched between 1/10/2005 and 1/11/2019 to identify dogs that underwent intracranial surgery for the resection of a meningioma. In order to be included, dogs needed to have had intracranial surgery performed to resect a mass lesion that was subsequently histopathologically confirmed to be a meningioma. Each patient needed to have complete medical records for the time that they were hospitalized. Dogs were excluded from the study if the medical records were incomplete or unavailable for review.

All dogs underwent general physical and neurological examinations by a board-certified veterinary neurologist or resident in a veterinary neurology training program. Information retrieved and analysed from the medical records included signalment (age, breed and sex), history and neurological examination findings, MRI findings and other imaging findings (thoracic and/or abdominal staging) if performed. The presence of intraoperative or postoperative complications, administration of medications (glucocorticoids and anti-epileptic medications), the number of days hospitalised and the survival to discharge were also recorded. The following variables were collected to allow statistical analysis of their effect on survival time; surgical approach (transfrontal, rostrotentorial or suboccipital), meningioma location, the use of an ultrasonic aspirator, the addition of intraoperative topical chemotherapy, the administration of oral hydroxyurea, the administration of post-operative radiotherapy and the meningioma histopathological subtype. The primary location of the meningioma was based on advanced imaging and was classified as being in either the olfactory lobe, frontal lobe, parietal lobe, temporal lobe, occipital lobe or the cerebellum.

All dogs were anaesthetized with protocols differing between referral centres and all magnetic resonance images were obtained with either 1.5 T imaging systems (Intera; Philips Medical Systems) or 0.4 T imaging systems (Aperto, Hitachi Medical Corporation). Owners were informed of the MRI findings and the treatment options were discussed with a board-certified veterinary neurologist or resident in a veterinary neurology training program. Surgical intervention was in the form of a craniotomy or craniectomy with the approach used dependent on the neuroanatomical localization of the meningioma. All surgeries were performed by a board-certified neurologist.

The use of an intraoperative ultrasonic aspirator was surgeon and institution dependent with three of the four included referral centres having access to an ultrasonic aspirator (Sonopet Ultrasonic Aspirator UST-2001 Miwatec Co., Ltd., Kawasaki, Japan; Integra, Cusa, Excel+ System; Sonocure Ultrasonic Aspirator., Tokyo Iken Co Ltd., Tokyo Japan). The use of intraoperative topical chemotherapy was surgeon dependent and was only utilised at one of the four referral centres included. This involved a single topical application of 5 mg of methotrexate and 100 mg of cytosine arabinoside onto the brain parenchyma following the surgical excision of the meningioma prior to closure. The recommendation of administering post-operative hydroxyurea was discussed and offered to all owners with the final decision being made by the owner, with all four referral centres utilising this adjunctive therapy for some dogs. The suggested dose of hydroxyurea was 50 mg/kg per os every other day. Five dogs received an initial daily loading dose for the first 7–14 days before the frequency was reduced to alternate days for the remainder of the treatment course.

The recommendation of post-operative radiotherapy was discussed and offered to all owners with the final decision being made by the owner. None of the included referral centres had the ability to perform radiotherapy on site and patients receiving radiotherapy were therefore referred to one of five other referral centres primarily dependent on their location. Radiation therapy was performed with a 4 or 6MV linear accelerator (Clinac 600c and 2100, Varian Medical Systems, Palo Alto, California, USA). Fractionation schemes used included the administration of 50Gy over 20 fractions, 48Gy over 12 fractions, 32 Gy over 4 fractions and 37 Gy over 5 fractions.

Survival time was defined as the period of time between the date of surgical resection of the meningioma and the recorded date of death or euthanasia. Information pertaining to whether the patient was alive or dead at the time of writing was attained by contacting the referring veterinary practice, not the owners. If the patient had deceased, the date of death was recorded and where possible, the cause of death was categorised as either neurological, non-neurological or unknown. For statistical evaluation, both ‘all cause’ mortality and cause specific mortality were used where appropriate.

### Statistical analysis

Statistical analysis was performed utilizing a standard statistical software package (SPSS). Kaplan Meier analysis was performed to attain the median survival times (95% confidence interval). Dogs still alive at the end of the study period were censored. Kaplan Meier analysis was used to assess the significance of seven different variables on survival time with factors compared pairwise over strata. Significance for all analyses was set at *P* < 0.05.

## Data Availability

The datasets used and/or analyzed during the current study are available from the corresponding author on reasonable request.

## Abbreviations

MRI: Magnetic Resonance Imaging
MST: Median Survival Time
